# Measurement of Cardiorespiratory Fitness in Children from Two Commonly Used Field Tests After Accounting for Body Fatness and Maturity

**DOI:** 10.2478/hukin-2014-0010

**Published:** 2014-04-09

**Authors:** Michael J. Hamlin, Meegan Fraser, Catherine A. Lizamore, Nick Draper, Jeremy P. Shearman, Nicholas E. Kimber

**Affiliations:** 1 Department of Social Science, Parks, Recreation, Tourism and Sport, Lincoln University, Christchurch, New Zealand.; 2 School of Sciences and Physical Education, University of Canterbury, Christchurch, New Zealand.; 3 School of Applied Sciences and Allied Health, Christchurch Polytechnic Institute of Technology, Christchurch, New Zealand.

**Keywords:** VO2_max_, aerobic performance, endurance, cardiorespiratory fitness, children

## Abstract

Body fat and maturation both influence cardiorespiratory fitness, however few studies have taken these variables into account when using field tests to predict children’s fitness levels. The purpose of this study was to determine the relationship between two field tests of cardiorespiratory fitness (20 m Maximal Multistage Shuttle Run [20-MST], 550 m distance run [550-m]) and direct measurement of VO2_max_ after adjustment for body fatness and maturity levels. Fifty-three participants (25 boys, 28 girls, age 10.6 ± 1.2 y, mean ± SD) had their body fat levels estimated using bioelectrical impedance (16.6% ± 6.0% and 20.0% ± 5.8% for boys and girls, respectively). Participants performed in random order, the 20-MST and 550-m run followed by a progressive treadmill test to exhaustion during which gas exchange measures were taken. Pearson correlation coefficient analysis revealed that the participants’ performance in the 20-MST and 550-m run were highly correlated to VO2_max_ obtained during the treadmill test to exhaustion (r = 0.70 and 0.59 for 20-MST and 550-m run, respectively). Adjusting for body fatness and maturity levels in a multivariate regression analysis increased the associations between the field tests and VO2_max_ (r = 0.73 for 20-MST and 0.65 for 550-m). We may conclude that both the 20-MST and the 550-m distance run are valid field tests of cardiorespiratory fitness in New Zealand 8–13 year old children and incorporating body fatness and maturity levels explains an additional 5–7% of the variance.

## Introduction

Cardiorespiratory fitness is considered an important marker of health in young people (Ruiz et al., 2009), and clear links have been established between the levels of fitness and coronary heart disease risk factors in adolescents and young children (Boreham et al., 2001). The most widely used indicator of cardiorespiratory fitness is the volume of oxygen that is consumed at maximal physical exertion (VO_2max_). Cardiorespiratory fitness can be objectively and accurately measured through laboratory tests such as progressive run or cycle tests to exhaustion, however, due to a number of disadvantages including high cost, the necessity of sophisticated equipment, availability of trained technicians and time constraints, these tests are impractical in population-based studies and for applied use. Alternatively, field-based tests do not usually require sophisticated equipment or trained technicians, and are relatively inexpensive, time efficient and easily administered to large numbers of subjects at the same time.

Two common field tests for the determination of cardiorespiratory fitness in children are the 550-m timed run/walk and the “Maximal Multistage 20-m Shuttle Run Test” (20-MST). The 20-MST was developed by Leger and Lambert (1982) as an alternative to the more traditional distance run test and has several practical advantages (Leger and Lambert, 1982). The 20-MST can be administered in a small area, difficulties with children pacing themselves are avoided, and the test is incremental and maximal intensities are only reached towards the last stages of the test. Alternatively, distance run tests like the 550 m run remain popular for school children as there is no specialised equipment required, large numbers of children can be tested together, and time required to conduct the test is short.

For a field test to be valid it is required to accurately and reliably measure what it claims to measure (Gore, 2000). Field-based fitness tests which commonly use predictive linear regression equations traditionally suffer from low relative validity when compared to a criterion measure (i.e. the “gold standard” measurement; for example, the criterion measure of cardiorespiratory fitness is VO_2max_ measured from respiratory gas exchange by indirect calorimetry). The validity of using the time to complete the 550 m (∼600 yard) run/walk test as a predictor of VO_2max_ has produced conflicting results with a review by Safrit (1990) reporting correlation coefficients ranging from 0.23 for 10 year old females to 0.72 for 9 year old males (Safrit, 1990). Considerable variation also exists in the reported validity of the 20-MST as a measure of VO_2max_ in children and adolescents with correlation coefficients ranging from 0.45 to 0.87 (Boreham et al., 1990]; Chia et al., 2005 (Table 4)). However, rarely do such studies account for body fatness and maturity when calculating these associations.

It is well accepted that body fatness influences fitness levels in adults (Mattila et al., 2007). Similarly, increased body fat is likely to lead to a decrease in performance in children, particularly where body weight is lifted or carried over distance (i.e. running) (Olds et al., 2007). Maturation also has a significant effect on children’s aerobic performance with treadmill endurance tests and finishing times in distance runs both demonstrating improvement with age from about 6 years up to approximately 14 years (Houlsby, 1986). Similar trends exist for lab-measured VO_2max_ levels in children which tend to increase in boys and girls up to puberty (Bar-Or, 1983). However, few reports investigating the validity of cardiorespiratory-type field tests in children have taken body fat and maturity levels into consideration in their calculations (Chia et al., 2005; Metz and Alexander, 1970; Safrit, 1990; van Mechelen et al., 1986). Those that have included these confounding variables used skinfolds as estimates of body fatness (Krahenbuhl et al., 1977; Mota et al., 2002), which can be less valid in children and adolescents (Rodriguez et al., 2005), or did not measure VO_2max_ directly (Mota et al., 2002). Therefore, the purpose of the present study was to determine the relationship between two commonly used cardiorespiratory field tests (550 m distance run, 20-MST) and direct measurement of VO_2max_ in children, after adjustment for body fatness (determined by bioelectrical impedance which has been found to be valid and reliable in children (Lim et al., 2009)) and maturation levels.

## Material and Methods

### Participants

Fifty-three participants (25 male, 10.6 ± 1.1 years, 142.9 ± 10.4 cm, 36.6 ± 8.4 kg body mass, 16.6 ± 6.0% body fat and 28 female, 10.6 ± 1.3 years, 143.9 ± 8.6 cm, 36.9 ± 7.1 kg body mass, 20.0 ± 5.8% body fat) volunteered to participate in the present study. Participants were informed of the tests’ protocols and procedures, and to ensure equal effort in all tests, encouragement was kept constant. Participants were familiarised with all tests prior to the study. The study was approved by the Lincoln University Human Ethics Committee and conformed to the standards set by the Declaration of Helsinki. Informed voluntary written consent was obtained from each participant and their parents prior to the start of the study.

### Procedures

The three test trials were conducted as separate sessions interspersed between 5–6 days for each individual. The field tests (550-m run and the 20-MST) were conducted as randomized balance trials and the VO_2max_ test was conducted only after the participants had completed their two field tests. All testing was completed within 3–4 weeks and all testing times were standardized to within 1 h to reduce any effect of diurnal variation. Testing was completed under similar climatic conditions (temperature, 18.5 ± 2.1 °C) at the participants’ school either on a calibrated running track around a grassed field (550-m run) on a concrete surface (20-MST), or in a private room set up for the VO_2max_ test. In a real world ecologically valid scenario participants ran in groups (12–15 for the 550 m run and 8–9 for the 20-MST) and were given similar encouragement to perform maximally in all tests. Participants wore shorts and a light T-shirt for the tests and were required to wear the same running shoes for all running trials although height and body mass were measured in bare feet. A standardized warm-up for the 550-m run and 20-MST consisted of 3 min of light jogging (about 7 km·h^−1^ pace) and 3 min of directed stretching of the lower limbs.

### Measures

Participants who were students at the same school, walked and/or ran as fast as they could without stopping until completing a distance of 550 m around a circular grassed track. Participants completed the test in age groups and were encouraged to walk or run at a pace sustainable for the entire distance. Participants could slow down but if they stopped for more than five seconds the examiner ended the test. The 550 m time was recorded manually on a stopwatch (S120-4020; Seiko, Tokyo, Japan) to the nearest tenth of a second and converted to average speed (distance run/time taken).

The 20-MST followed previously described protocols (Leger and Lambert, 1982) where participants ran back and forth between two lines set 20 m apart. Participants commenced running at an initial speed of 8.5 km·h^−1^, which increased by 0.5 km·h^−1^. The pace was established by an audio signal emitted from a compact disc (20-m Shuttle Run test CD, Australian Sports Commission). Participants were given a warning if they did not reach the line in time with the audio signal, and the test was stopped if the participant could not reach the line for two successive shuttles or if the participant stopped voluntarily. Completed shuttles were converted to total distance covered (number of completed shuttles × 20 m) and velocity at exhaustion (speed from the last completed shuttle) for statistical analysis

The treadmill test was conducted in a private room at the school (temperature, 19.9 ± 1.9 °C barometric pressure 1009 ± 8.7 mbar) in similar running attire to the field testing. Each participant warmed up on the treadmill (Rodby™, RL 1600E, Enhorna, Sweden) for 3 min at 8.0 km·h^−1^ and then completed 3 min of stretching of the lower limbs. The protocol for the test was the 2-min modified Balke treadmill test as described previously (Rowland, 1993) with constant speed and increasing elevation designed specifically for paediatric populations with varying fitness levels. Initial treadmill starting speed and elevations were; poorly fit 4.8 km·h^−1^ and 6 %, sedentary 5.2 km·h^−1^ and 6%, active 8.0 km·h^−1^ and 0%, athlete 8.4 km·h^−1^ and 0%. Thereafter, the speed was held constant but treadmill elevation increased every 2 min by either 2% (poorly fit and sedentary participants) or 2.5% (active and athlete participants). Participants initial fitness levels were calculated from the results of the 20-MST. The test was stopped when the participant could not maintain the required pace or had reached voluntary exhaustion. Because of the inherent difficulty of using adult criteria to determine maximal oxygen consumption in children (Rowland, 1993), we used the highest attained VO_2_ value at the point of volitional fatigue as the VO_2max._

Ventilatory and expired gases were measured breath-by-breath using a portable gas exchange system (MetaMax® 3B; Cortex Biophysik, Leipzig, Germany). Before testing, the gas analyser was calibrated for volume (Hans Rudolph 5530 3 L syringe; Kansas City, MO, USA) and gas composition (15% O_2_ and 5% CO_2_). Oxygen consumption (VO_2_), minute ventilation (V_E_) and respiratory exchange ratio (RER) were measured. Paediatric face masks (Hans Rudolph, Kansas City, MO, USA) with small dead spaces were fitted to participants allowing simultaneous breathing at the mouth and nose. To de-emphasise breath-to-breath variation, values for V̇O_2_ were smoothed by taking the average for every 15-second time period. Heart rate was recorded continuously by means of a heart rate monitor (S610; Polar, Kempele, Finland) in all participants during the treadmill test and a subgroup of 7 participants during the field testing. Anthropometric measurements were taken at school by one research assistant. Standing and sitting height as well as leg length was measured with a portable stadiometer. Two measurements were made to the nearest 0.1cm. If these measurements differed by more than 0.5cm, a third measurement was taken and the mean of the closest two measurements was recorded. Maturity status was determined from anthropometric measures in accordance with research by Mirwald et al. (2002). Bioelectrical impedance analysis (BIA) was also performed using dual-frequency hand and foot electrodes (MF-BIA2; InBody 230, Biospace, Seoul, Korea), participants were instructed to empty their bladders before measurement. Children were not asked to fast prior to BIA, however to minimise the influence of hydration status on BIA measures they were instructed to hydrate appropriately prior to testing. The analyser uses an alternating current of 250 mA at frequencies of 20 and 100 kHz. Body mass, fat mass, and percent body fat are estimated from prediction equations developed for European participants.

### Statistical Analysis

The Statistical Analysis System (Version 9.3, SAS Institute, Cary NC) was used to calculate means and standard deviations for the various performance measures from each of the three tests. Residuals from the SAS output were initially checked for normality. We used the magnitude-based inferences approach (Hopkins, 2002) because small performance changes can be beneficial (Hopkins et al., 1999) and are sometimes not considered when using conventional statistics. Differences in variables between gender groups were determined using unpaired t-tests with an alpha level of 0.05. Uncertainty in the estimate of changes was presented as 90% confidence intervals. Pearson correlations were computed between selected variables to provide an indication of overall association between the testing protocols. We used Cohen’s (Cohen, 1988) guidelines for classifying the correlations (i.e. r < 0.30, small; r = 0.31–0.50, moderate; r > 0.50, large). In addition, a multivariate multiple stepwise regression was performed on the log-transformed data to determine the amount of variance that could be attributed to body fat and maturation levels. Finally, a Fisher transformation was used after averaging the correlation coefficients found from previously published validation studies to adjust for any skewness of the sampling distribution when averaging all correlation results; p values were reported for those who do not adhere to magnitude-based inference testing.

## Results

Table 1 shows the participants’ performance data obtained from the laboratory and field testing. Compared to girls, the boys attained a substantially higher VO_2max_ after accounting for body mass (3.3 ± 2.7 ml.kg·min^−1^, mean difference ± 90% confidence limits). Boys were also more likely to complete more shuttle laps (9.4 ± 8.1 shuttle laps), gain more distance (183 ± 150 m) and attain a higher velocity at exhaustion (0.5 ± 0.4 km·h^−1^) in the 20-MST test, and were likely to perform better in the 550-m run test (−14.4 ± 8.2 s, 1.1 ± 0.6 km·h^−1^ for the run time and average velocity, respectively) compared to girls. In a subset of 7 participants, we found no clear differences in HR_max_ between the 3 different exercise tests (199 ± 11, 204 ± 17, 208 ± 10 for the treadmill, 20-MST and 550 m run respectively, p = 0.12 – 0.65).

Large to very large correlations were found between treadmill VO_2max_ and performance in the respective fitness tests (Table 2). Correlations were slightly different between boys and girls for the same performance comparisons, however all correlation coefficients remained large to very large.

To determine the amount of variance that could be attributed to body fat levels and maturation when predicting VO_2max_ from either the 20-MST or 550-m run a multivariate multiple stepwise regression was performed. The only substantial improvement came from the addition of the body fat percentage data, which accounted for additional 5–7% of the regression variance (Table 3). Accounting for body fat percentage and maturation increased the total correlation coefficient between VO_2max_ and 20-MST for boys and girls combined from 0.70 to 0.73. Similarly, the correlation coefficient between VO_2max_ and 550-m run increased from 0.59 to 0.65 after accounting for body fat percentage and maturation.

## Discussion

The aim of this study was to determine the relationship between the 20-MST, 550-m run and direct measurement of VO_2max_ after accounting for body fatness and maturation in children. As previously reported (Dencker et al., 2007) the males in the current study had substantially higher aerobic ability (measured either by treadmill VO_2max_, 20-MST or 550-m run) than females. There were no substantial differences in body height, body mass or age between the males and females, however; females possessed substantially more fat mass. Children’s VO_2max_ scores reported in this study compare well to similar-aged children from previous studies (Krahenbuhl et al., 1977; van Mechelen et al., 1986).

Both the 550 m run and the 20-MST field test demonstrated acceptable levels of validity (i.e. r > 0.50 according to the Cohen’s scale) when compared to the criterion measure (VO_2max_), suggesting both field tests are suitable for providing adequate estimations of cardiorespiratory fitness. Additionally, we found that accounting for body fatness in particular, increased the association between the field tests and the lab-based VO_2max_. This suggests that estimations of cardiorespiratory fitness. incorporating a measure of body fatness into the field test measures would help improve the estimation of cardiorespiratory endurance (VO_2max_) from such tests. Older and larger children tend to have more fat, particularly girls (Rowland, 1990), but also higher oxygen consumption capacity (Bar-Or, 1983), therefore accounting for the confounding effects of body fatness and maturation in children during field-based aerobic performance testing is likely to get a more precise indication of their actual VO_2max_.

After accounting for body fatness and maturation the validity coefficient for cardiorespiratory fitness measured by the 550 m run was 0.65 for males and females combined, which indicates a large positive correlation according to Cohen’s cut-offs (Cohen, 1988) and is similar to some previously reported research (Krahenbuhl et al., 1977; Metz and Alexander, 1970; Safrit, 1990). However, what is also obvious from these studies is the large variation in the validity coefficients between studies for the association between the 550 m run and VO_2max_ (Table 4). To estimate the overall effect of the association between the 550 m run and VO_2max_ from these studies we calculated the average overall validity coefficient (after adjustment using the Fisher transformation). We found that even after incorporating the large variations in the reported validity of the 550 m run, there was a positive correlation (0.51) between performances in the 550 m run and VO_2max_ attained either by bicycle erogometry or treadmill testing. Variation in the validity correlation may be due to a number of factors including motivation, an inability of the children to pace themselves during the test, altered environmental factors between the field and the laboratory testing and lack of experience in distance running (Krahenbuhl et al., 1978).

When maturation and body fatness are controlled for the validity coefficient of the 20-MST and VO_2max_ in the current study (0.73) is consistent with previous research (Table 4). Again there is some inconsistency in the validity coefficient between the 20-MST and VO_2max_ reported between studies, however, the overall average (0.62) suggests a strong and positive correlation between 20-MST performance and VO_2max_. In this case, the variation in the correlation of these two parameters is likely to be attributed to the factors mentioned above including motivation, inexperience and measurement error of the different tests, but not the inability to pace since the test is self-paced through the timed audio signals.

Adjusting for maturation levels alone failed to significantly improve the regression variance for VO_2max_ and either 20-MST or 550 m run. Maturation probably failed to contribute because most of the subjects were of similar maturation levels with a total range of 10.6 ± 1.2 years (mean ± SD). A larger cross-section of children from a more varied age range would be required to investigate the effect of maturation in more depth.

Overall, taking the combined results of boys and girls, the 20-MST had a higher correlation with VO_2max_ compared to the 550 m run. We suggest this may be due to differences in testing procedures of the two field tests. The 550 m run is a timed effort where the students use self-motivation and pacing skills to complete the distance. In contrast, the 20-MST is not influenced by pacing strategy since children are required to keep in time with the audio signals. Indeed the 20-MST (a progressive workload test) is very similar in nature to the modified Balke treadmill test used in this study, and may explain the higher correlations between the two measures.

A major concern with field-based testing in children is the issue of motivation. There are tight guidelines around assessment of VO_2max_ on a treadmill or cycle ergometer that ensures the subject has worked to the same endpoint (i.e. volitional exhaustion), however such controls are not in place in field-based testing. To ensure children were working at similar exercise intensities during the 3 different fitness tests we measured heart rate in a subset of 7 participants. Maximal heart rate was not substantially different between the 3 fitness tests indicating that the children in this study were exercising at similar intensities in all 3 tests.

A limitation of this study is the fact that all participants were from one school and were homogenous in terms of age and body size. Therefore, the substantial correlations that were found are applicable to this group only and a greater sample size from a wider cross-section of children from different age and socioeconomic groups would be required to confirm these finding before applying the results to the general population.

## Conclusions

We conclude that the 550 m run and the 20-MST are both valid field tests for cardiorespiratory fitness in 8–13 year-old children. Results from either field test will give a good indication of children’s maximal aerobic power. Moreover, including body fat levels into the estimation improves the associations between these two field tests and direct measurement of VO_2max_.

## Figures and Tables

**Table 1:** Performance and physiological responses in the modified Balke treadmill, 20-MST and 550-m run tests

	Males (n = 25)	Females (n = 28)	Total (n = 53)
Treadmill test			
V̇O_2max_ (L·min^−1^)	1.8 ± 0.5	1.7 ± 0.4(0.32)	1.8 ± 0.4
V̇O_2max_ (ml·kg^−1^·min^−1^)	49.3 ± 4.4	46.1 ± 6.4(0.04)^*^	47.6 ± 5.8
RER	1.0 ± 0.1	1.0 ± 0.1(0.70)	1.0 ± 0.1
HR_max_ (b·min^−1^)	201.6 ± 9.0	205.6 ± 7.2(0.11)	203.9 ± 8.2
Time to exhaustion (s)	571.8 ± 160.1	556.4 ± 161.1(0.76)	562.6 ± 158.8
20-MST			
Laps	42.9 ± 19.1	33.4 ± 16.0(0.05)^*^	38.0 ± 18.0
Distance covered (m)	860 ± 364	677 ± 313(0.05)^*^	765 ± 347
Velocity at exhaustion (km·h^−1^)	11.0 ± 1.0	10.5 ± 0.9(0.05)^*^	10.7 ± 0.9
550-m run			
Run time (s)	154.0 ± 14.4	168.3 ± 19.1(0.00)^*^	161.9 ± 18.5
Average velocity (km·h^−1^)	12.9 ± 1.2	11.9 ± 1.3(0.00)^*^	12.4 ± 1.4

Data are mean ± SD (and between gender p values). HR_max_, maximal heart rate, Time to exhaustion, time taken to reach exhaustion; Laps, number of ‘shuttles’ completed during the 20-MST; Distance covered, total distance that was completed during the 20-MST; Velocity at exhaustion; velocity reached just prior to exhaustion during the 20-MST; Run time, time taken to complete the 550-m run distance; Average velocity, average velocity over the entire 550-m run.

* Substantially different from males.

**Table 2:** Association between the laboratory and field tests.

	Treadmill vs. 20-MST	Treadmill vs. 550 run	20-MST vs. 550 run
Total	0.70 ± 0.13	0.59 ± 0.17	0.72 ± 0.12
Males	0.59 ± 0.25	0.67 ± 0.21	0.71 ± 0.19
Females	0.79 ± 0.14	0.50 ± 0.26	0.68 ± 0.19

Pearson correlations ± 90 confidence intervals were calculated on V̇O_2max_ (ml·kg·min^−1^) for the treadmill, distance covered (m) during the 20-MST, and average velocity (km·h^−1^) over the 550 m run. All correlations were substantial and significant.

**Table 3:** Results of the multiple regression analysis of VO_2max_ with 20-MST, 550 m run, body fat percentage and maturation level.

	R	R^2^	SEE (%)
20-MST			
Basic model	0.70	0.490^*^	0.01
Body fat%	0.73	0.527^*^	0.11
Maturation	0.71	0.511	0.46
Body fat% × Maturation	0.73	0.535^*^	0.46
550-m run			
Basic model	0.59	0.349^*^	0.52
Body fat%	0.65	0.418^*^	0.13
Maturation	0.60	0.355	0.46
Body fat% × Maturation	0.65	0.418^*^	0.55

* p < 0.05

**Table 4:** Validity of the 550 m run and the 20-MST in children.

	Correlation coefficient	Sample (n/sex/age)	Criterion Test
*550 m run*			
Doolittle and Bigbee (1968)	0.62	9/ M/ 14–15 yr	Bicycle
Safrit (1969)	0.70	20/ M/ 11 yr	Treadmill
	0.70	20/ M/ 10 yr	Treadmill
	0.71	20/ M/ 9 yr	Treadmill
	0.67	20/ F/ 11 yr	Treadmill
	0.23	20/ F/ 10 yr	Treadmill
	0.41	20/ F/ 10 yr	Treadmill
Metz and Alexander (1970)	0.27	30/ M/ 14–15 yr	Treadmill
	0.66	30/ M/ 12–13 yr	Treadmill
Krahenbuhl et al. (1977)	0.42	48/ M&F/ 8 yr	Treadmill
	0.57	20/ M/ 8 yr	Treadmill
Cureton et al. (1977)	0.62	196/ M&F/ 7–12 yr	Treadmill
Hamlin et al. (current study)	0.59	53/ M&F/ 8–13 yr	Treadmill
**Average**	**0.50**		
*20-MST*			
Van Mechelen et al. (1986)	0.76	82/ M&F/ 12–14 yr	Treadmill
Leger et al. (1988)	0.71	188/ M&F/ 8–19 yr	extrapolation
Boreham et al. (1990)	0.87	51/ M&F/ 14–16 yr	Treadmill
Liu et al. (1992)	0.69	48/ M&F/ 12–15 yr	Treadmill
Mahoney (1992)	0.83	10/ M/ 12 yr	Treadmill
	0.76	10/ F/ 12 yr	Treadmill
Barnett et al. (1993)	0.72	55/ M&F/ 12–17 yr	Treadmill
McVeigh et al. (1995)	0.85	18/ F/ 13–14 yr	Treadmill
	0.68	15/ M/ 13–14 yr	Treadmill
Matasuzaka et al. (2004)	0.80	132/ M&F/ 8–17 yr	Treadmill
Aziz et al. (2005)	0.68	8/ M/ 17 yr	Treadmill
Chia et al. (2005)	0.45	41/ M/ 16 yr	Treadmill
Hamlin et al. (current study)	0.70	53/ M&F/ 8–13 yr	Treadmill
**Average**	**0.62**		

Sample: n/sex/age; M, males; F, females (Aziz et al., 2005; Barnett et al., 1993; C. A. G. Boreham et al., 1990; Chia et al., 2005; Cureton et al., 1977; Doolittle and Bigbee, 1968; Krahenbuhl et al., 1977; L. A. Leger et al., 1988; Liu et al., 1992; Mahoney, 1992; Matsuzaka et al., 2004; McVeigh et al., 1995; Metz and Alexander, 1970; Safrit, 1990; van Mechelen et al., 1986)
